# L-Asparaginase-Induced Continuous Hyperglycemia With Type 1 Diabetes-Related Antibodies and HLA Genotypes: A Case Study

**DOI:** 10.7759/cureus.30067

**Published:** 2022-10-08

**Authors:** Yasuhisa Furuta, Shigeru Yatoh, Hitoshi Iwasaki, Yoko Sugano, Motohiro Sekiya, Hiroaki Suzuki, Hitoshi Shimano

**Affiliations:** 1 Department of Endocrinology and Metabolism, Faculty of Medicine, University of Tsukuba, Tsukuba, JPN; 2 Community-Based Medicine System, Faculty of Medicine, University of Tsukuba, Tsukuba, JPN; 3 Internal Medicine, Toride Community Medical Education Station, University of Tsukuba Hospital / Toride-Kitasoma Medical Association Hospital, Toride, JPN; 4 International Institute for Integrative Sleep Medicine, University of Tsukuba, Tsukuba, JPN; 5 Life Science Center for Survival Dynamics, Tsukuba Advanced Research Alliance, University of Tsukuba, Tsukuba, JPN; 6 Agency for Medical Research and Development-Strategic Basic Research Program (AMED-CREST), Japan Agency for Medical Research and Development, Chiyoda-ku, JPN

**Keywords:** acute lymphoblastic leukemia (all), type 1 diabetes mellitus (t1d), l-asparaginase, hla, anti-gad antibody

## Abstract

A 19-year-old male presented with fatigue and dyspnea on exertion. He was diagnosed with acute T-cell lymphoblastic leukemia. After following the Group for Research on Adult Acute Lymphoblastic Leukemia (GRAALL) 2003 protocol that incorporates L-asparaginase (L-Asp) treatment, blood glucose levels became elevated for more than one year and insulin secretion was depleted. Anti-glutamic acid decarboxylase (GAD) and anti-islet antigen 2 (IA-2) antibody levels were both positive, which is rare. The patient’s HLA genotype was sensitive for type 1 diabetes. L-Asp can cause transient hyperglycemia as a side effect. However, cases with the anti-GAD antibody have not been reported in L-Asp-induced diabetes. In summary, L-Asp-induced continuous hyperglycemia might be associated with a type 1 diabetes-related HLA genotype through elevations of anti-GAD and anti-IA-2 antibodies.

## Introduction

L-asparaginase (L-Asp) is used as a therapeutic drug for acute T-cell lymphocytic leukemia [1,2]. Its side effects include hyperglycemia, pancreatitis, and dyslipidemia [3-8]. L-asparaginase-associated hyperglycemia has been reported in 4-20% of pediatric patients receiving Escherichia coli-derived asparaginase for acute lymphoblastic leukemia and in 4-17% of patients receiving Erwinia-derived asparaginase [9]. It can cause diabetic ketoacidosis due to severe insulin deficiency [10,11].

Type 1 diabetes is caused by the progressive autoimmune destruction of pancreatic beta cells producing insulin. The anti-glutamic acid decarboxylase (GAD) antibody is present in 70-80% of patients with new-onset type 1 diabetes [11].

The type 1 diabetes susceptibility HLA-alleles, DRB1*0405, DRB1*0901, DRB1*0802-DQB1*0302, DRB1*0405-DQB1*0401, and DRB1*0901- DQB1*0303, were described in patients with anti-GAD antibody and insulin deficiency in a Japanese population [12,13].

No cases with the anti-GAD antibody have been reported in L-Asp-induced diabetes. Here, we report the first case of L-Asp-induced diabetes, together with the transient appearance of anti-GAD antibody, associated with the type 1 diabetes susceptibility HLA alleles, DRB1*04:05/*08:02.

## Case presentation

A 19-year-old male experienced fatigue and dyspnea on exertion for two months. His blood count revealed leukocytosis, erythrocytopenia, and thrombocytopenia. As a result of a bone marrow biopsy, he was diagnosed with acute T-cell lymphoblastic leukemia. 

The Group for Research on Adult Acute Lymphoblastic Leukemia (GRAALL) 2003 protocol is outlined in Table 1 [14]. Remission induction therapy, including L-Asp and a large amount of corticosteroid, was initiated. L-asparaginase was only used during the first remission induction therapy. After five weeks, a bone marrow test showed hematological remission.

**Table 1:** GRAALL 2003 Protocol GRAALL: Group for Research on Adult Acute Lymphoblastic Leukemia

Remission induction therapy protocol		
Medication	Amount	Day
Daunorubicin	85 mg (50 mg/m^2^)	1–3
	51 mg (30 mg/m^2^)	15,16
Vincristine	2 mg	1,8,15,22
L-asparaginase	10,000 U (6,000 U/m^2^)	8,10,12,20,22,24,26,28
Cyclophosphamide	1,250 mg (750 mg/m^2^)	1,15
Prednisolone	100 mg (60 mg/m^2^)	1–14
Consolidation therapy protocol		
Medication	Amount	Day
Cytarabine	6,100 mg (4,000 mg/m^2^)	1,2
Dexamethasone	20 mg	1,2
L-asparaginase	15,000 U (10,000 U/m^2^)	3

Consolidation therapy was then started. The fasting plasma glucose level increased to over 400 mg/dL. Selected biochemical test results are shown in Table 2. Plasma levels of insulin and C-peptide were 2.0 μU/mL and 0.25 ng/mL, respectively. Anti-GAD and anti-islet antigen 2 (IA-2) antibody levels were measured at 13.4 U/mL and 3.1 U/mL, respectively, and samples were therefore considered positive for these two antibodies.

**Table 2:** Laboratory Findings WBC: white blood cells, RBC: red blood cells, Hb: hemoglobin, Ht: hematocrit, Plt: platelets, PT: prothrombin time, APTT: activated partial thromboplastin time, IRI: immunoreactive insulin, CPR: C-peptide immunoreactivity, GAD antibody: glutamic acid decarboxylase antibody, IA-2 antibody: islet antigen 2 antibody, ALB: albumin, AST: aspartate aminotransferase, ALT: alanine aminotransferase, LDH: lactate dehydrogenase, γ-GTP: gamma-glutamyl transferase, T-Bil: total bilirubin, IP: inorganic phosphorus, BUN: blood urea nitrogen, CRE: creatinine, CRP: C-reactive protein

Lab Parameters	Value	Unit	Lab Parameters	Value	Unit
WBC	900	/μL	ALB	3.8	g/dL
RBC	291 x 10^4^	/μL	AST	18	U/L
Hb	8.8	g/dL	ALT	64	U/L
Ht	25.9	%	LDH	106	U/L
Plt	8.5 x 10^4^	/μL	γ-GTP	28	U/L
			T-Bil	1.3	mg/dL
PT	15.6	s (INR 1.33)	Na	130	mEq/L
APTT	40.9	s (con 26.9 s)	Cl	95	mEq/L
			K	4.5	mEq/L
Glucose	483	mg/dL	Ca	9	mg/dL
IRI	2	μU/mL	IP	4.7	mg/dL
CPR	0.25	ng/mL	BUN	18.4	mg/dL
GAD antibody	13.4	U/mL	CRE	0.49	mg/dL
IA-2 antibody	3.1	U/mL	CRP	0.09	mg/dL

Intensive insulin therapy was started on a sliding scale. The total amount of insulin given was approximately 60 units per day. An insulin secretory defect was thought to cause hyperglycemia. L-asparaginase induces diabetes mellitus as a side effect [14], and both anti-GAD and anti-IA-2 antibody levels were found to be positive. Since this was not common, we regularly measured C-peptide and anti-GAD and anti-IA-2 antibody levels during the intensive insulin therapy.

The time courses for measured glucose, insulin, and C-peptide levels are shown in Table 3. The fasting C-peptide level was found to gradually recover. The total amount of insulin administered decreased from approximately 60 units to about two units per day.

**Table 3:** Time courses of glucose, insulin, and C-peptide L-asp: L-asparaginase, FPG: fasting plasma glucose, CPR: C-peptide immunoreactivity, GXR: insulin glargine

Months after L-asp and prednisolone administration	months	1	7	9	11	13
FPG	mg/dL	483	108	80	90	124
CPR	ng/mL	0.25	0.54	0.8	0.66	1.35
CPR index	-	0.052	0.5	1	0.73	1.1
Total amount of daily insulin	units/day	63	16	20	2	3
Long-acting insulin analog on the day before	units/day	0	GXR 9	GXR 8	GXR 3	GXR 3

**Figure 1:**
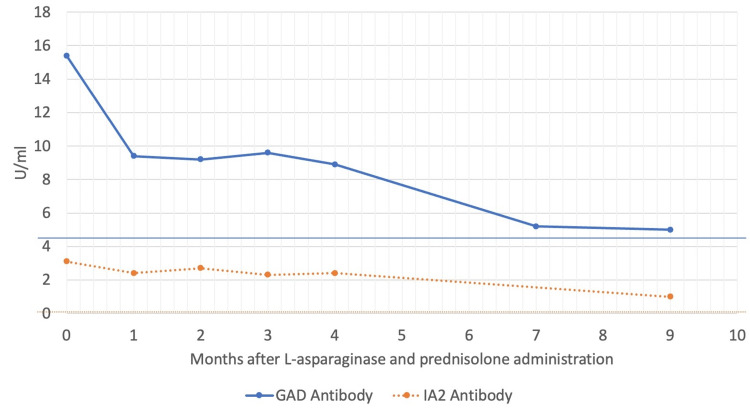
Time courses for glutamic acid decarboxylase (GAD) and islet antigen-2 (IA-2) antibody levels

Time courses for GAD and IA-2 antibodies are shown in Figure 1. Both anti-GAD and anti-IA-2 antibody levels decreased over time. The anti-GAD antibody level was considered negative nine months after remission induction therapy was initiated.

In order to evaluate insulin secretion 11 months after remission induction therapy was started, a glucagon test was performed and plasma glucose/insulin/C-peptide levels before and after breakfast, as well as urinary C-peptide, were assessed. These findings are shown in Table 4. Insulin secretion did not become depleted.

**Table 4:** Evaluation of insulin secretion, 11 months after remission induction therapy Administer two units of insulin glargine the previous day. Eat 20% of 2,000 kcal/day meals. PG: plasma glucose; IRI: immunoreactive insulin; CPR: C-peptide immunoreactivity; CPI, C-peptide index: CPR/PG*100 Urinary CPR 36 μg/day

Glucagon test	minutes	0	3	6	10	20	30
PG	mg/dL	201	220	238	247	265	280
IRI	μU/mL	8.4	22.7	15.4	12.2	7.9	5.7
CPR	ng/mL	1.88	2.92	2.76	2.53	2.22	1.84
Meal test	PG	IRI	CPR	CPI			
(Unit)	(mg/dL)	(μU/mL)	(ng/mL)				
Before breakfast	159	6.4	1.74	1.09			
After breakfast	226	12.8	2.38	1.05			

**Figure 2:**
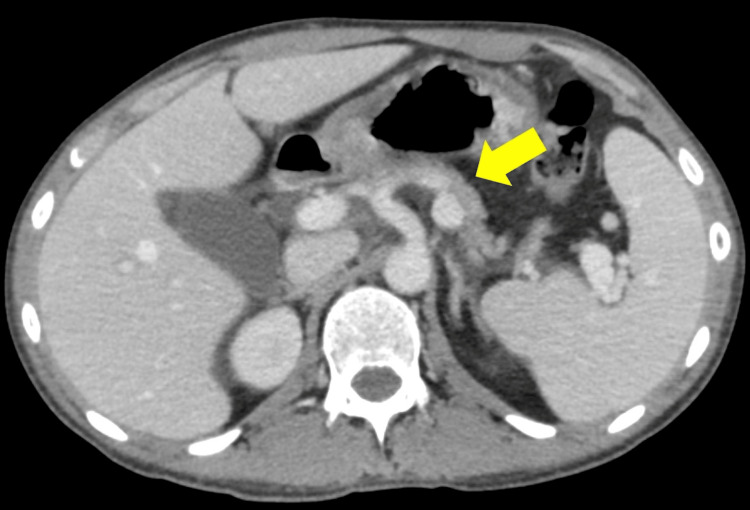
Abdominal plain computed tomography scan The yellow arrow indicates an atrophic pancreas.

The patient was administered because of a relapse of acute T-cell lymphoblastic leukemia. He felt a loss of appetite and fatigue; such stresses subsequently induced hyperglycemia. A computed tomography (CT) scan showed atrophy of the pancreas (Figure 2). Therefore, the pancreatic exocrine function was evaluated. A fecal digestion status test showed fatty stool. A low pancreatic function diagnostic test score of 20.6% (standard: >73.4%) was obtained.

Furthermore, an HLA gene test was performed for a marrow transplant. Incidentally, the patient’s HLA-DRB1, consisting of 04:05 and 08:02, was found to be a susceptible gene for type 1 diabetes [15].

## Discussion

L-asparaginase is an enzyme that hydrolyses asparagine to aspartic acid and ammonia, causing a depletion of asparagine in cells. Insulin incorporates three molecules of asparagine; therefore, L-Asp can inhibit its synthesis in pancreatic beta cells to cause transient hyperglycemia and diabetes mellitus [16,17]. The percentage of cases with acute lymphoblastic leukemia in whom hyperglycemia occurs due to L-Asp with steroid therapy is 2.5-23% [18]. Hyperglycemia occurs within 5-10 days after the initiation of L-Asp therapy. Although patients require transient insulin therapy, hyperglycemia improves in almost all cases.

In addition to transient glucose intolerance, some rare cases develop pancreatic diabetes with L-Asp-related pancreatitis. Twenty-one percent of patients with L-Asp-related pancreatitis require insulin therapy in the acute phase and 6% of patients continue insulin therapy for one year [19,20]. However, no L-Asp-related diabetes mellitus cases have been positive for both anti-GAD and anti-IA-2 antibodies. 

When we explored a mechanism for how the antibodies had appeared, it became clear the patient had specific HLA genes that made him susceptible to type 1 diabetes mellitus. The relative risk of this genetic combination was estimated to be 15 times [15]. It may be that the patient’s condition made him more susceptible to developing type 1 diabetes mellitus. L-asparaginase subsequently induced pancreatic beta-cell impairment, leading to the leakage of intracellular substances into the blood. As a result of antigen presentation, anti-GAD and anti-IA-2 antibody levels became transiently positive.

Next, we considered how the increase in these antibodies affected the decrease in insulin due to the impairment of pancreatic beta cells.

In Table 3, C-peptide levels seemed to be recovering gradually. The recovery might be mainly due to the resolution of glucotoxicity and functional beta cell impairment induced by L-Asp. However, the results of the glucagon test and assessment of plasma glucose/insulin/C-peptide levels before and after breakfast indicated hyperglycemia and insufficient C-peptide. In spite of the hyperglycemia, urinary C-peptide was only 36 μg/day (Table 4). To assess these results comprehensively, the significantly decreased insulin level after L-Asp administration gradually recovered. However, the plasma glucose level was not normal. We considered that insulin secretion had decreased compared to before L-Asp administration.

We discussed whether the limited decrease of insulin depended on the L-Asp effect or autoimmune destruction of pancreatic beta cells due to type 1 diabetes mellitus. Compared to a typical course of L-Asp-induced diabetes, it was uncommon that pancreatitis did not occur clinically and insulin therapy was necessary after a year. We, therefore, speculated that the mechanism involved was due to type 1 diabetes mellitus. However, we thought that the speculation was unlikely because type 1 diabetes mellitus usually causes extensive destruction of beta cells and needs the administration of massive insulin permanently. On the other hand, the limited decrease in insulin was explainable if L-Asp had caused pancreatitis. The atrophic pancreas in the CT scan and the decrease in pancreatic exocrine function were consistent with post-pancreatitis changes. We might have missed an acute phase of pancreatitis.

## Conclusions

We describe a unique case of L-asparaginase-induced continuous hyperglycemia with type 1 diabetes-related antibodies. Although diabetes mellitus occurred after the administration of L-Asp, it was found that anti-GAD and anti-IA-2 antibodies, which are indicators of type 1 diabetes mellitus, were transiently high. This could be explained by the presence of a type 1 diabetes mellitus-sensitive HLA gene.

It is our firm belief that HLA genotype testing should be performed when the anti-GAD antibody is detected and hyperglycemia persists after L-Asp administration.
